# Plaque removal effectiveness of 3D printed dental hygiene chews with various infill structures through artificial dog teeth

**DOI:** 10.1016/j.heliyon.2022.e09096

**Published:** 2022-03-12

**Authors:** Su Hyun Lee, Hyun Woo Kim, Hyun Jin Park

**Affiliations:** Department of Biotechnology, College of Life Science and Biotechnology, Korea University, Anam-dong, Seongbuk-gu, Seoul, 02841, Republic of Korea

**Keywords:** 3d food printing, Pet food, Dental hygiene chew, Plaque removal efficacy, Infill density

## Abstract

Pet food has recently been in the spotlight as an auxiliary approach to manage oral health, since it helps dogs or cats to take relatively simple care of their mouths at home. Especially, dental hygiene chew is crucial to remove teeth accumulation or plaque by chemical or mechanical methods. This study applied 3D printing to dental chews, which should be tailored to dogs’ individual tooth structure and preferences. The optimum methods for making dental hygiene chews based on corn starch with glycerin for extrusion-based 3D printing were developed. The viscoelasticity of dental chews increased with increasing glycerin content. According to the infill level (40%, 60%, or 80%) and glycerin content, texture and plaque removal efficacy were investigated using a texture analyzer and dog dentures. A 60% infill level with 10% and 20% glycerin content had the best plaque removal efficacy in both canines and premolars. A lattice structure design with square holes was more effective for canines, whereas a crumbly texture was more effective for premolars.

## Highlights

• Starch-based dental chew ink was formulated with various glycerin concentrations.
• The rheology of dental chew ink was dependent on the addition of glycerin.
• Increasing glycerin content up to 20% contributed to improved printing performance.
• Printed objects had higher breaking force but less hardness than control.
• Plaque removal efficacy was enhanced by infill densities.

## 1 Introduction

Periodontal disease is one of the most common oral disorders in animal dental hygiene, especially in small dog breeds (Niemiec, 2012). It has been reported that approximately 80% of dogs older than two years experience oral pain, infection, or inflammation resulting from periodontal disease (Harvey et al., 1994). However, since dog guardians cannot communicate directly with their dogs, regular observation and management by their guardians are important in understanding the oral condition of dogs (Lewis et al., 2005). The oral daily care of dogs can be managed using tartar removal through scailing by going to the veterinary clinics or at home each 6–12 months (Bellows et al., 2019). Regular toothbrushing or other methods, guardians can effectively manage their dog's oral care. Gorrel and Rawlings (1996) reported that a combination of tooth brushing and dental chews reduces both the likelihood of developing gingivitis and accumulation of dental deposits relative to that with toothbrushing alone every other day. Dental chew effectiveness in reducing plaque or tartar build-up on dog teeth has already been investigated by many researchers (Bjone et al., 2005; Clarke et al., 2011).

The effects of dental chews can be broadly divided into chemical and physical methods. First, dental chews may improve oral health through chemical reactions by adding functional ingredients, including polyphosphates, pantothenic acid, riboflavin, or other effective components to prevent gingivitis (Stella et al., 2018). Dental chews help control plaque and tartar buildup through mechanical abrasions, such as removing food stuck between teeth (Schroeder and Attström, 1979). However, to date, most studies have observed only the chemical effects of dental chewing, and there are few studies on its mechanical properties.

In terms of texture, most dental chew products require a hard texture that can be repeatedly chewed to achieve their plaque-removing effect. For hard textures, products on the market are generally created using injection molding or extrusion (Sanderson, 2021; Mateo et al., 2020). However, these mass-produced dental chews have several drawbacks owing to their limited model design and internal structure. These factors make these chews less effective at removing plaque because factors such as dog size, individual chewing behavior, and time spent chewing can also influence dental chew effectiveness (Carroll et al., 2020). It is hard for dental chews to fit each dog's oral cavity structure due to using molds of the same size, making dental chess less effective at removing plaque. Nevertheless, a few studies have investigated the relationship between anatomical differences, such as tooth size or cavity structure, and the size and shape of various chews (Harvey, 2002; Noh et al., 2017). In addition, the necessary hardness of the chew for each dog relies on many factors, such as breed and age, which require customization. Moderate hardness is needed for repeated chewing, but if the chew is too hard or smooth, it has the potential to cause tooth fractures or pose a choking risk. Some studies have reported the efficacy of abrasive chewing dental hygiene treatments in improving oral health (Brown and McGenity, 2005). However, there are few solutions for modifying the texture or hardness above extrusion-type limits.

Three-dimensional (3D) printing for food has become one of the latest innovations in food design and manufacturing, with potential research and industrial applications. Printing technology enables the creation of new shapes of food products, providing freedom in structure, texture, and taste (Feng et al., 2019; Pallottino et al., 2016). Using this technology, the hardness or structure of foods can be modified, which is one of the main advantages of using 3D printing. Many studies have been conducted on modeling structures with infill patterns and the densities of 3D printed objects. For instance, the hardness or fracturability of products can be modified by modulating infill patterns and densities (Gué nard-Lampron et al., 2021; Huang et al., 2019). Despite its potential, 3D printing has not yet been applied in the pet food industry, especially in feeds and snacks. Nevertheless, printing may be a notable breakthrough for customizing dental chew products for pets. 3D printing allows limitless control of the infill structure of dental chew products and may raise the efficacy of plaque removal by changing the model design or size. Moreover, it is useful for customizing ingredient compositions, including flavor and nutrient content, on demand according to each dog's preference.

In this study, we developed a starch-based dough as a dental chew ink model with different glycerin contents for printing. Additionally, this study aimed to build several dental hygiene chew models with different infill levels and carry out a comprehensive study of the relationship between the structure and texture of dental chews and dog teeth by imitating the chewing process using a texture analyzer.

## 2 Materials and methods

### 2.1 Materials

Soy protein isolate, agar, and maltodextrin were purchased from ES Food Materials (Gyeonggi-do, Korea). Corn starch was purchased from Tureban Co. (Gyeonggi-do, Korea). Corn syrup was purcahsed from Ottogi Co., Ltd (Gyeonggi-do, Korea). Glycerin was purchased from Duksan Pure Chemicals (Gyeonggi-do, Korea). Original petite dental chews (Mars Petcare US, Franklin, Tennessee, USA) were used as controls for texture analysis and plaque removal effectiveness tests.

### 2.2 Food-ink preparation

To investigate the effect of glycerin on the dimensional stability of the dough, as shown in Table 1, the relative weight of glycerin (0%, 10%, 20%, and 30%) was replaced with distilled water to prepare the dental dough.

**Table 1:** Dental chew dough formulation for printing.

Ingredients (g)		Sample			
		Gly 0%	Gly 10%	Gly 20%	Gly 30%
Powder	Corn starch	30	30	30	30
	Agar	5	5	5	5
	Maltodextrin	4	4	4	4
	Soy protein isolate	5	5	5	5
Liquid	Glycerin	0	10	20	30
	Corn syrup	6	6	6	6
	Water	50	40	30	20
Total		100	100	100	100

Glycerin and distilled water were stirred using a magnetic stirrer (HS 15-26P, MiSung Scientific Co., Ltd., Korea) for 15 min. Corn syrup (6 g) was added to the liquid, and the mixture was stirred for 15 min. While stirring, corn starch (30 g) and soy protein isolate (5 g) were premixed in a powder. Agar (5 g) and maltodextrin (4 g) were added to a powder mixture and mixed homogeneously. The stirred liquid was then poured into a powder mixture. The dough was kneaded manually and placed in a refrigerator. Finally, the samples were sealed in a zipper bag and placed in a refrigerator at 4 °C, kept at room temperature for 30 min, and transferred to a plastic 50-mL syringe before printing and other tests.

### 2.3 Rheological properties of dough

The dynamic viscoelastic properties of dough were observed using a controlled stress rheometer (Paar Physica MCR 302, Anton Paar, Austria) with a diameter of 25 mm (PP25/S) and a gap of 1.3 mm at 25 °C. All samples loaded in the rheometer were rested for 15 min before starting the measurement to allow the collapsed internal tissues to be restored. Cooking oil was coated on the sample edges to prevent the surface from drying out during the waiting time. Strain sweep tests were performed at 10 rad/s to obtain the linear viscoelastic region. Angular amplitude sweep tests were conducted at 1% strain with a frequency range of 0.1–100 rad/s to measure the storage modulus (G′) and loss modulus (G″) of the samples.

### 2.4 3D food printing behavior

**Figure 1:**
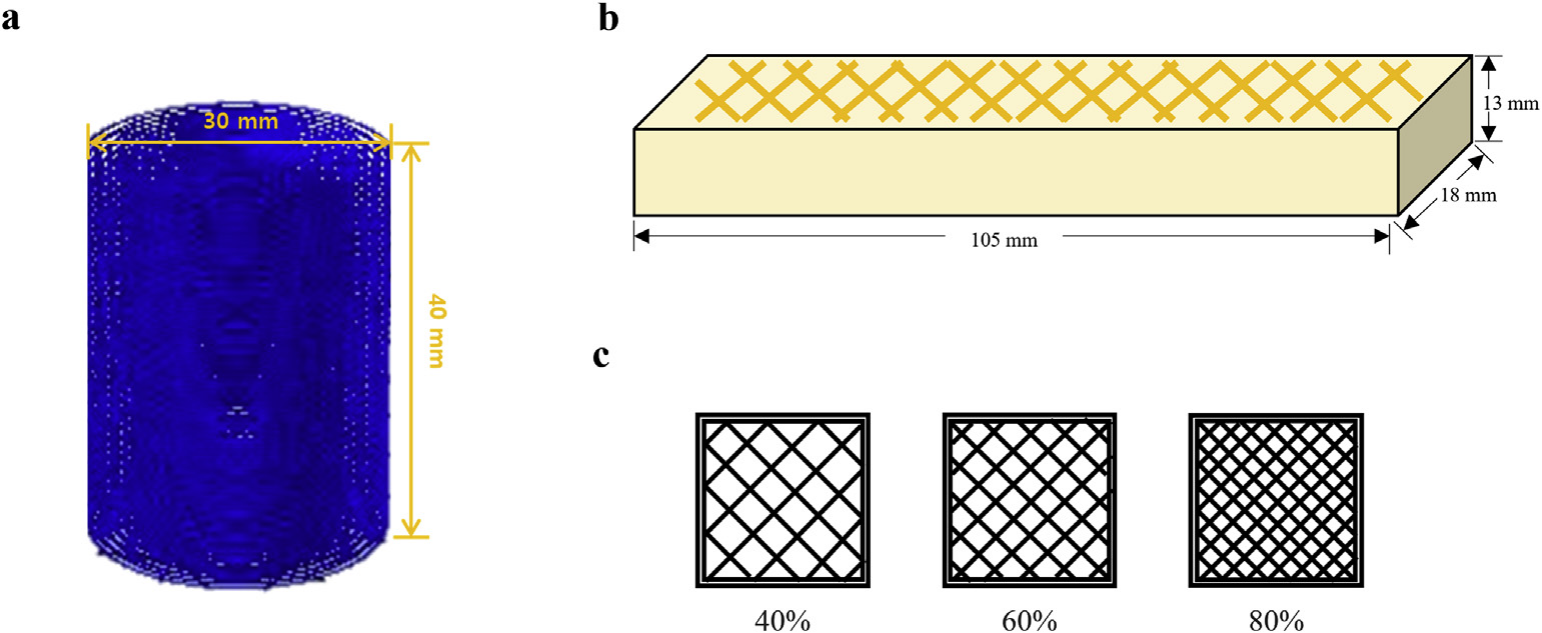
Model design of printing behavior (a: 3D model design for printing behavior, b: 3D model design of dental chew, c: 3 types of infill pattern - 40%, 60% 80%).

3D printing was conducted using a 3D food printer (YOLI-LAB, YOLILO Co., Ltd., Korea). A cylindrical shape with a diameter of 30 mm and height of 40 mm was utilized to observe the printing behavior of the dental chew dough (Figure 1a). The nozzle size for printing was 1.1 mm. The printing parameters were set as follows: layer height, 1.1 mm; first layer height, 1.7 mm; and nozzle speed, 40 mm/s. Printability and solidity were calculated according to the methods described by Azam et al. (2018) and Kim et al. (2021) with slight modifications. The heights of the printed objects were measured using a digital caliper (BD500-150; Bluetec, Seoul, Korea). The two values were calculated using Eq. (1) and Eq. (2):

(1) $$\text{Printability ​}\left(\text{\%}\right)=100\times \frac{\text{achieved ​height ​}\left(\text{mm}\right)}{\text{target ​height ​of ​the ​template ​}\left(\text{mm}\right)}$$

(2) $$\text{Dimensional ​stability ​}\left(\text{\%}\right)=100\times \frac{\text{ ​height ​of ​object ​after ​}1\text{ ​h ​ ​}\left(\text{mm}\right)}{\text{achieved ​height ​}\left(\text{mm}\right)}$$

### 2.5 Making dental chews

#### 2.5.1 Dental chew printing (identifying infill density)

The formulations suitable for printing were selected. The dental chew model was designed as a cuboid shape with dimensions of 18 × 13 × 105 mm (Figure 1b). For the internal lattice structure, the rectilinear infill pattern of Slic3r was used with infill densities of 40%, 60%, and 80% for each treatment, as shown in Figure 1c. The samples were printed with two perimeters and one solid bottom layer. The printing parameters were set as follows: layer height, 1.6 mm; first layer height, 2.0 mm; and nozzle speed, 40 mm/s. The nozzle size used was 1.5 mm.

#### 2.5.2 Post-processing of printed objects

Printed objects were first steamed for 40 min after printing to gelatinize the corn starch. After steaming, the samples were cooled immediately in a refrigerator at 4 °C for 20 min. Subsequently, the samples were hot air-dried at 75 °C in a dry oven for 4 h. After removing dental chew samples from the dry oven, they were wrapped to prevent drying until the texture analysis or plaque removal efficacy test.

### 2.6 Texture analysis of dental chew

**Figure 2:**
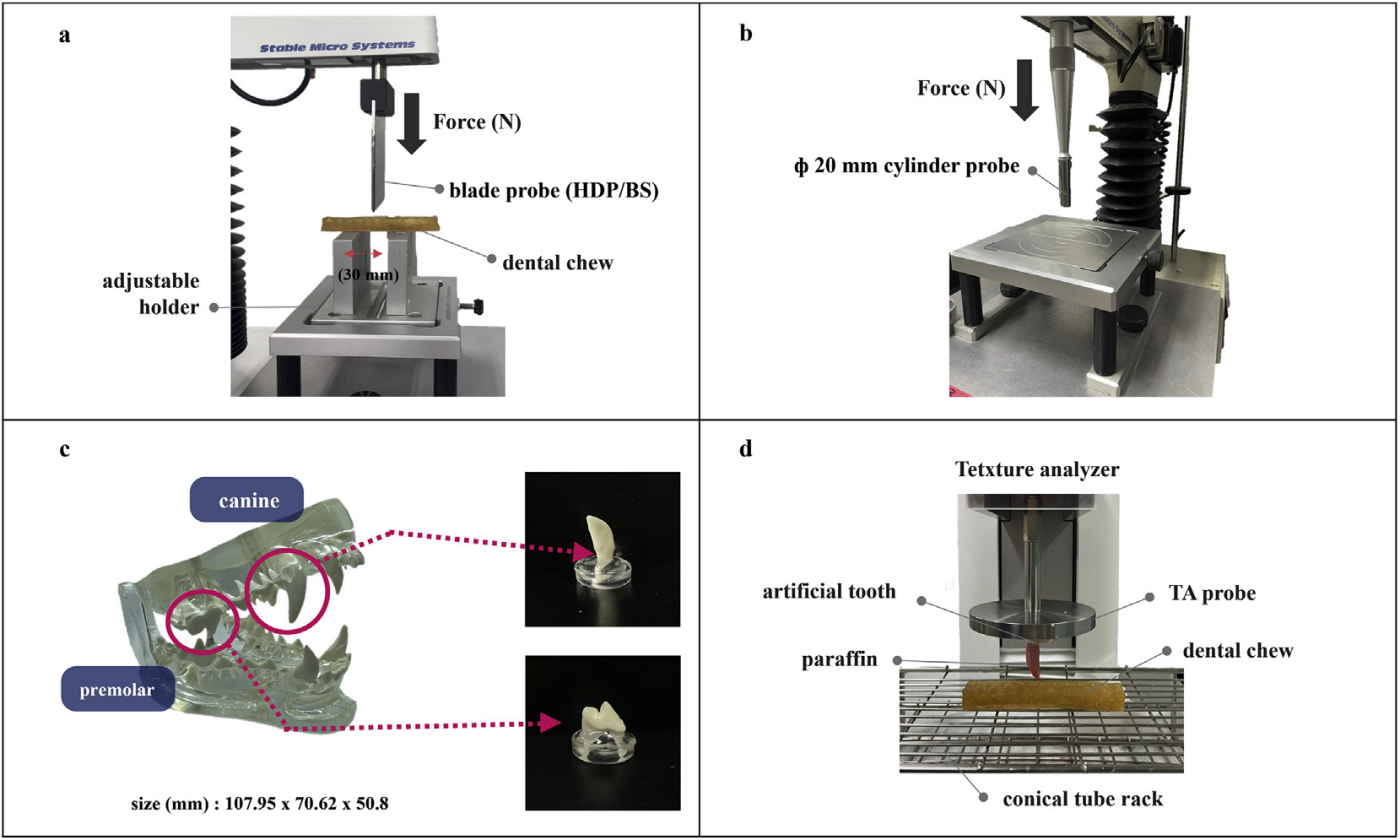
Texture analysis and plaque removal efficacy test design (a: puncture test, b: three point bending test, c: dog dentures and probe, and d: plaque removal efficacy test).

Mechanical characterization of dental chews was performed using a texture analyzer (TA-XT plus 50, Stable Micro Systems, UK) with a slight modification of the method described by He et al. (2020). Commercial products were used as the controls. The texture analyzer settings used for the puncture and three-point bending tests were carried out at a test speed of 1.00 mm/s and pre/post speed of 5.00 mm/s, using a trigger force of 0.05 N. As shown in Figure 2a, the breaking force (N) and fracturability (mm) were evaluated using a three-point bending test. A blade probe (HDP/BS) was used with a base plate gap of 30 mm. A puncture test was conducted to determine the hardness of dental chew products, as shown in Figure 2b. The ф 20-mm cylinder probe (P/20) was used for the test. The compression target distance was set to 7 mm.

### 2.7 Moisture content

The moisture content of the dental chew objects and controls was determined using a moisture analyzer (Model MX-50; A&D Co., Ltd., Seoul, Korea). Each sample was dried at a temperature of 130 °C until reaching a decrease in mass of 0.1%/min. The mass for each sample was 5 g.

### 2.8 Plaque removal effectiveness

The plaque removal efficacy was confirmed by scraping artificial plaque coated on a tooth surface and weighing the remaining amount. To evaluate the effectiveness of chewing on plaque removal, paraffin (Paraplast®, Leica Biosystems, St. Louis, USA) was used as artificial plaque. Paraffin wax was stained with red dye (ECO/REACH Tomato Red Dye #D23192, FRENCH COLOR & FRAGRANCE CO., INC., USA) for visualization. The right maxillary canine (#104 in the modified triadan system by Floyd (1991)) and left maxillary premolar (#208) were extracted from a small-medium breed dog typodont (9196 clear canine jaw, GPI Anatomicals, USA). The extracted teeth were embedded in resin, as illustrated in Figure 2c. The denture with resin (W_1_) was weighed. The tooth was then coated with melted paraffin wax at 70 °C. A plaque removal test was performed using a texture analyzer as shown in Figure 2d (Lamy TA-TX 700, Germany). The paraffin-coated tooth (W_2_) was attached to a compression probe (ф 50 mm). The dental chew was placed in a test tube rack. The probe was downed at 5 mm/s in compression mode to imitate chewing. The scratched, paraffin-coated teeth (W_3_) were weighed. Eq. (3) was used to calculate the effect of plaque removal:

(3) $$\text{Plaque ​removal ​efficiency ​}\left(\text{\%}\right)=\frac{W_{2}-W_{3}}{W_{2}-W_{1}}\times 100$$

### 2.9 Data analysis

All measurements were repeated three times for each step. SPSS (SPSS 25.0, IBM, Chicago, IL, USA) was used for statistical analyses. The significance of treatment effects was estimated using one-way analysis of variance (ANOVA) and Duncan's range test (P < 0.05).

## 3 Results and discussion

### 3.1 Rheological properties of dough

**Figure 3:**
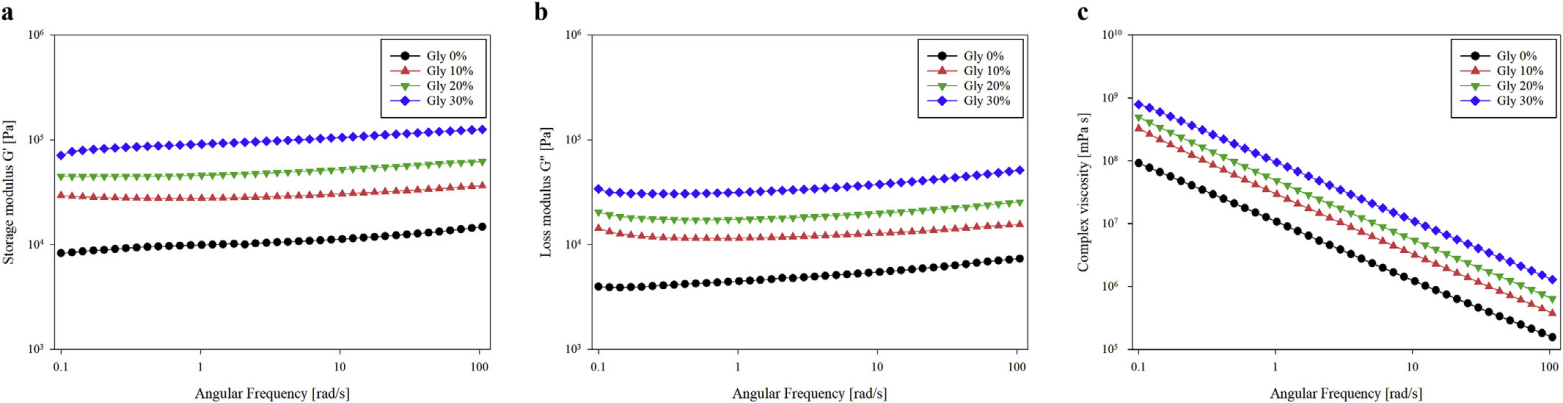
Effect of different glycerin levels on the dynamic rheological properties of dental chew pastes; Storage modulus (G′) (a), loss modulus (G″) (b), and complex viscosity (η∗) (c); () Gly 0%, () Gly 10%, () Gly 20%, () Gly 30%.

The viscoelastic properties of the dental chew pastes are presented in Figure 3. All samples exhibited solid-like behavior because the storage modulus (G′) was higher than the loss modulus (G″), as shown in Figure 3a and b. This result is beneficial for maintaining the stability of the shape after printing. This indicates that it is suitable for self-support after deposition on the printer surface (Jeon et al., 2021; UribeWandurraga et al., 2020). Figure 3c shows that the complex viscosity (η∗) decreased when the frequency (ω) increased in all samples. This indicates shear-thinning behavior (Jo et al., 2021). Shear-thinning behavior is associated with flow through during extrusion and lack of shape deformation after printing (Pérez et al., 2019).

With the increasing concentration of glycerin, the complex viscosity, G′ and G″ values, also increased for a given oscillatory frequency, which means that the addition of glycerin enhanced both the elasticity and viscosity of the dough complex system. This means that the storage and loss moduli increased significantly upon the addition of increasing amounts of glycerin, despite the same amount of other solid content. This is possibly due to the much higher viscosity of glycerin. Each sample was prepared with a different proportion of glycerin instead of water. Decreasing the moisture with increasing glycerin would lead to higher stickiness, resulting in increased G′ and G″. Based on the viscosity properties, it has been proven that dough ink can be smoothly extruded and is suitable for printing.

### 3.2 3D printing behavior

**Figure 4:**
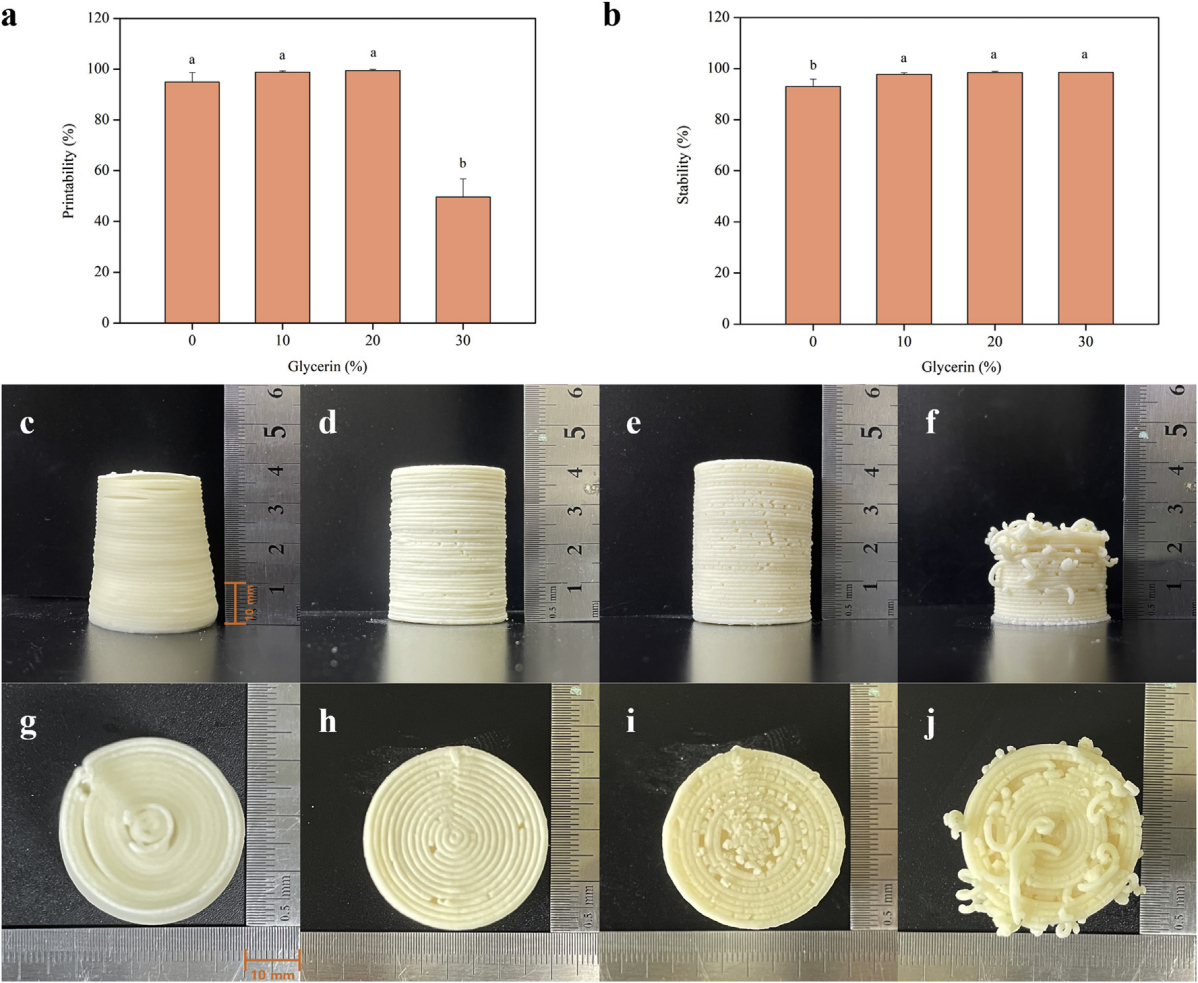
Printability (a), stability (b), and images of 3D printed dental chew dough with different glycerin concentrations (c,g: Gly 0%, d,h: Gly 10%, e,i: Gly 20% and f,j: Gly 30%).

Figure 4 shows the printing behavior of the paste in relation to the glycerin content. Figure 4a shows the printability, and Figure 4b shows the shape stability for 1 h after printing. The maximum printability of the target structure was best achieved using glycerin 20% (99.4%), followed by 10% (98.9%), 0% (94.9%), and 30% (49.6%). Conversely, the maximum stability 1 h after printing was exhibited using glycerin 30% (98.5%), followed by 20% (98.4%), 10% (97.7%), and 0% (93.0%). When the glycerin content was higher, the samples experienced more discontinuous extrusion, creating broken threads. Visually, all dough formulations, except glycerin 30%, presented shape fidelity with the model. When using glycerin 30%, as presented in Figure 4f and j, extrusion stopped while printing the 23rd layer and did not achieve the targeted height. Meanwhile, the sample without glycerin had poor ability to resist hydrostatic pressure and collapsed slightly (Figure 4c and g). Therefore, it can be concluded that glycerin 10% and 20% dough are equally suitable for printing because there was no statistical difference in printability and stability.

This trend was confirmed by rheological properties. The dough with a higher G′ exhibited improved printing accuracy and gel strength in the printing results. The shape fidelity and stability were improved with the addition of glycerin within the printable range. Regarding 3D printing behavior, the thread in the sample with glycerin 30% was broken during printing. The formulation with 30% of glycerin was too sticky to print and make the object. From both the viscoelastic properties and printing performance results, dough with 10% and 20% glycerin were selected for subsequent experiments examining texture analysis and plaque removal efficacy according to infill levels.

### 3.3 Texture analysis for post-processing samples

**Figure 5:**
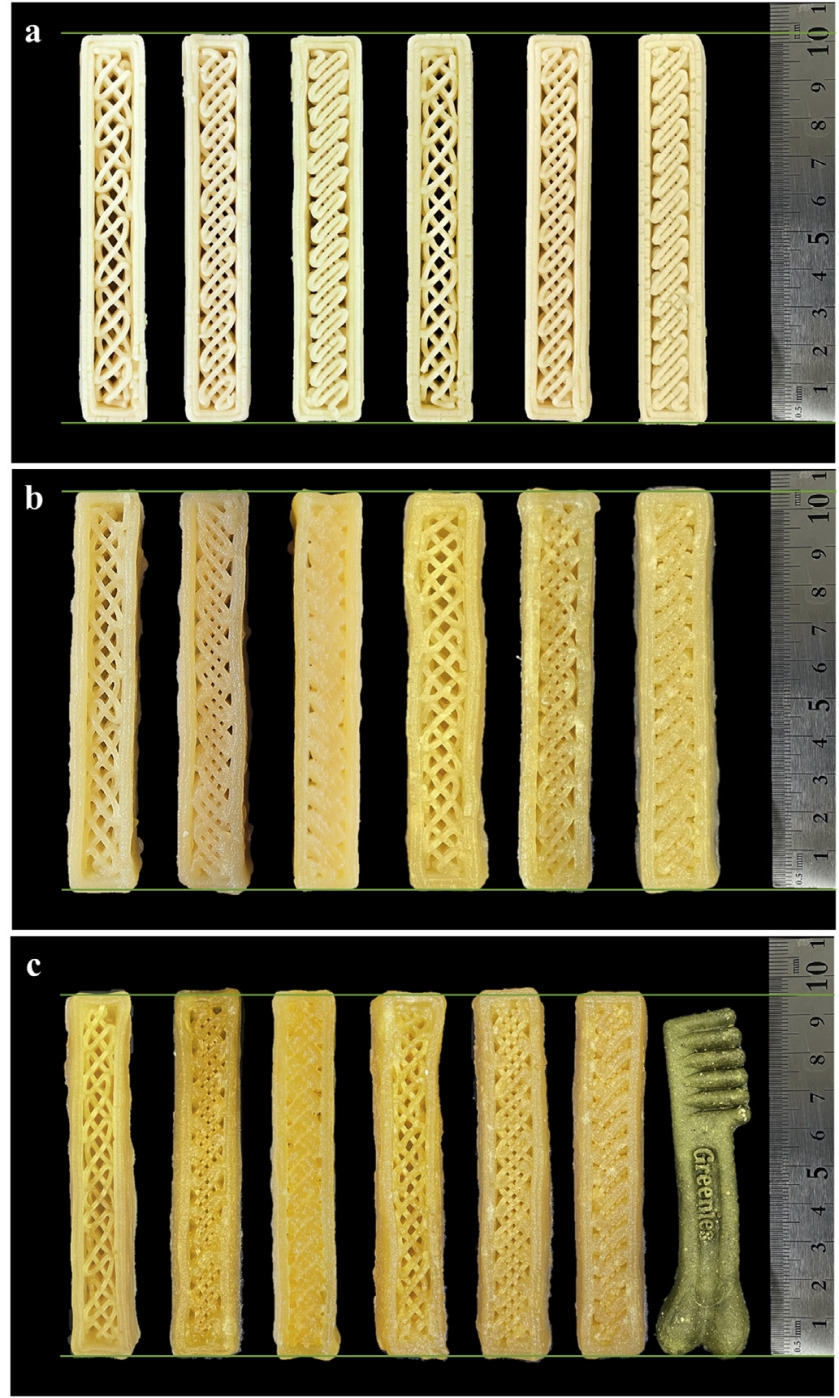
3D printed dental chew model design; printed alone objects (a), printed + steamed objects (b), printed + steamed + hot-air dried objects (c). Each figure from left to right: Gly 10%-infill 40% (G10-40), Gly 10%-infill 60% (G10-60), Gly 10%-infill 80% (G10-80), Gly 20%-infill 40% (G20-40), Gly 20%-infill 60% (G20-60), Gly 20%-infill 80% (G20-80). Control (the rightmost object in c).

Figure 5 shows the visual appearance of the printed and post-processed (steaming and hot-air dried) objects with different infill levels (40%, 60%, and 80%). All samples maintained their resolution, especially with respect to the internal structure, after steaming and air drying.

Table 2 shows the textural characteristics of the post-processed dental chew objects. From the puncture test, hardness (N) was recorded as the maximum force on the force-distance graph. When the infill level increased, the hardness also increased for the same glycerin percentage. The correlation of strength with infill level is consistent with the results of other studies (Feng et al., 2020; Liu et al., 2018). The higher the infill level, the lower the porosity of the sample and the denser the structure of the sample. Therefore, greater force is required to puncture the denser sample structure. Regarding the glycerin concentration, glycerin 20% was less hard than glycerin 10% at the same infill level, respectively. This may be due to the water-holding properties of glycerin in the starch-water system. The glycerin enters between the corn starch polymers and prevents the polymers from sticking together strongly during steaming (Chen et al., 2017). Since more moisture was lost with glycerin 10% than with glycerin 20%, it may have affected the hardness results. However, compared to the control, all samples had much lower hardness, and it was inferred that the printed and post-processed objects had smoother textures.

**Table 2:** Texture analysis of dental treats by glycerin percentage and infill level; Gly 10%-infill 40% (G10-40), Gly 10%-infill 60% (G10-60), Gly 10%-infill 80% (G10-80), Gly 20%-infill 40% (G20-40), Gly 20%-infill 60% (G20-60), Gly 20%-infill 80% (G20-80).

Sample	Weight (g)	Moisture content (%)	Three-point bending test		Puncture test
			Breaking force (N)	Fracturability (mm)	Hardness (N)
G10-40	12.98 ± 0.46^e^	13.14 ± 0.06^e^	101.95 ± 14.67^b^	18.49 ± 3.33^a^	301 ± 8.72^de^
G10-60	15.87 ± 0.41^c^	14.88 ± 0.27^d^	108.11 ± 11.3^ab^	18.85 ± 1.48^a^	359.18 ± 20^b^
G10-80	17.11 ± 0.87^b^	20.44 ± 0.41^b^	125.2 ± 8.31^a^	16.88 ± 1.67^a^	403.51 ± 33.4^a^
G20-40	14.62 ± 0.21^d^	14.01 ± 0.54^de^	96.02 ± 13.29^ab^	18.51 ± 4.95^a^	275.54 ± 18.03^e^
G20-60	17.42 ± 0.05^b^	18.7 ± 2.01^c^	99.67 ± 12.38^b^	17.99 ± 1.12^a^	321.36 ± 13.6^cd^
G20-80	20.11 ± 0.04^a^	21.93 ± 0.33^a^	106.08 ± 9.95^ab^	14.31 ± 1.99^ab^	352.79 ± 9.96^bc^
Control	17.36 ± 0.11^b^	9.69 ± 0.11^f^	72.69 ± 5.36^c^	9.99 ± 0.68^b^	432.09 ± 13.5^a^

The data recorded are presented as the mean ± standard deviation (SD). Superscripts within columns indicate significant differences (p < 0.05) between values.

We obtained the breaking force and fracturability results of dental chews using the three-point bending test. The breaking force was defined as the peak force at which the product began to break, and the distance at that point was fracturability on the force-distance graph (Bajaj and Singhal, 2007). The breaking force increased with increasing infill level for the same glycerin content, similar to the results for hardness in the puncture test. Glycerin 10% had a higher breaking force than that of glycerin 20%. The similar trend in breaking force and hardness can be understood from the point of view that breaking force is often used as an indicator of sample strength (Chen et al., 2013; Esan et al., 2015).

In contrast, these trends were different from those of the control. The control had a higher hardness in the puncture test, but it had a lower breaking force in the three-point bending test. Moreover, fracturability was not significantly different between different infill densities or glycerin concentrations, but it was much higher than that of the control. This means that the control was much more brittle than the printed objects, so all samples had more chewiness than the control. This indicates that samples made by printing were chewier than the control, even though the control was more rigid than the samples.

In summary, when the structure of the samples was denser, both the breaking force and hardness increased. Samples with 10% glycerin were stiffer than those with 20% glycerin for the same infill level due to the characteristics of glycerin. Nevertheless, both glycerin 10% and glycerin 20% with all infill densities had lower hardness than the control, but they had higher breaking force and fracturability.

### 3.4 Evaluation of plaque removal efficacy

At present, many studies have investigated the effect of dental hygiene chews on plaque or tartar removal in vivo studies with dogs through feeding, but no research has investigated the removal efficacy by mimicking texture analyzer-based chewing. It is noteworthy to check plaque removal efficacy with artificial teeth covered with paraffin instead of real bacteria-based biofilm plaque from the point of view of examining the mechanical effect.

**Figure 6:**
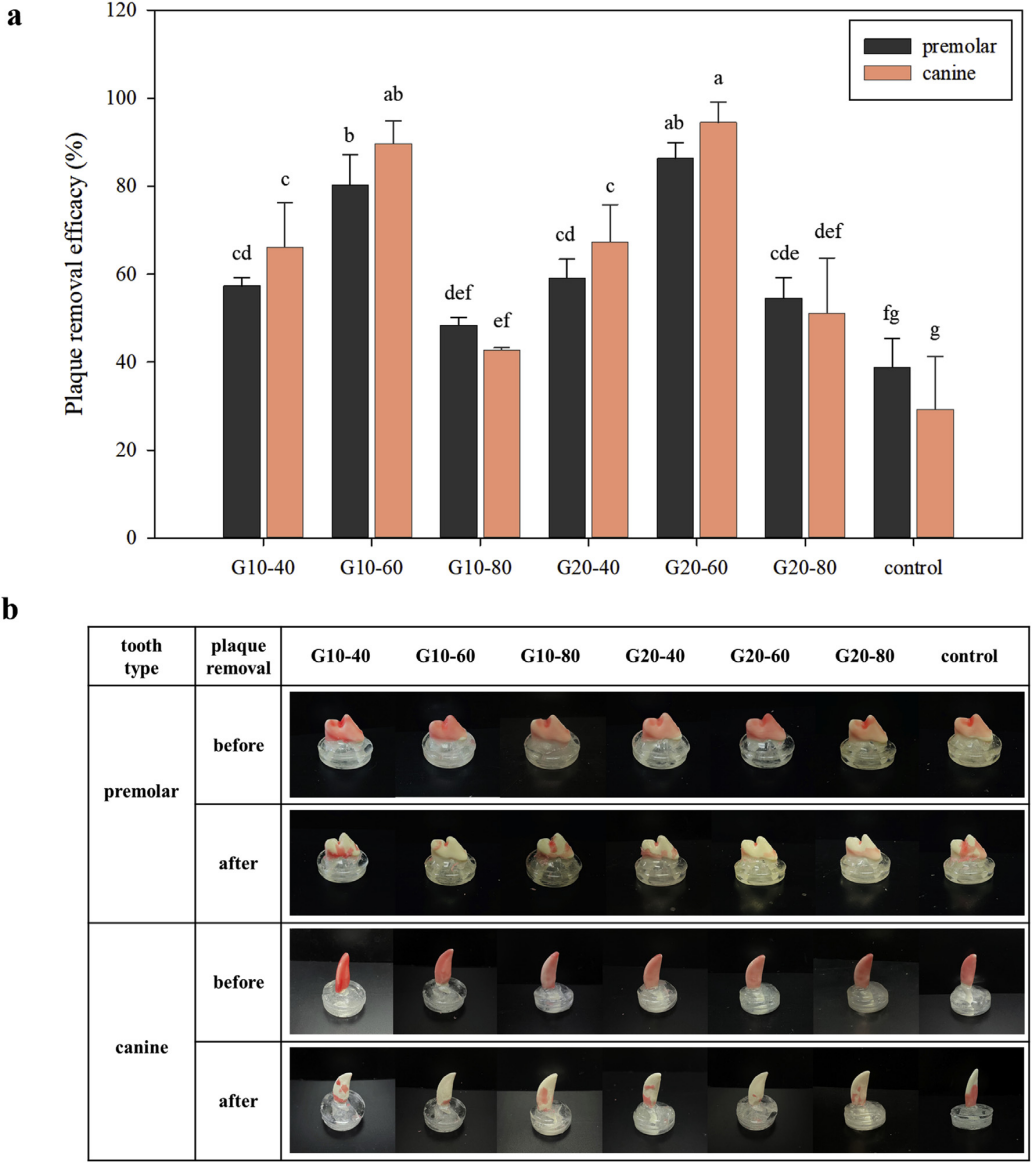
Plaque removal efficacy (%) for premolars and canines (a). Dental chew and paraffin-coated teeth (premolar and canine) before and after plaque removal test (b); Gly 10%-infill 40% (G10-40), Gly 10%-infill 60% (G10-60), Gly 10%-infill 80% (G10-80), Gly 20%-infill 40% (G20-40), Gly 20%-infill 60% (G20-60), Gly 20%-infill 80% (G20-80). The same small letter within bars indicates homogeneous groups established using ANOVA (p < 0.05).

Plaque removal efficacy was confirmed by using dog dentures to mimic the chewing process with a texture analyzer. Dentures with paraffin were placed into a square hole made with a rectilinear infill design inside the dental chew, and the coated paraffin was scraped off with the dental chew. Figure 6a shows the premolar and canine plaque removal efficacy of dental chews. The plaque removal efficacy of both glycerin 10% and glycerin 20% dental chew was higher than that of the control, and there was no significant difference between glycerin 10% and glycerin 20% chew. Considering infill density, dental chew at the 60% infill level was most effective in both canine and premolars, followed by 80% infill, 40% infill, and control. At the 40% and 60% levels, removal efficacy in canines was higher than in premolars, whereas, at 80% and control, the efficacy was higher for premolars. The difference in removal effectiveness for each tooth by infill level indicates that the efficacy according to the tooth dimensions or oral geometric structures, which vary depending on the breed and age, can be improved through infill density control.

Generally, more paraffin is removed from the canine than from the premolar. This might be due to the geometrical difference between the canine and premolar, as shown in Figure 6b, which has two cups that are relatively equal in size with a deep developmental groove between them. This deep groove may affect the efficacy of plaque removal.

Overall, the experimental dental chews were more effective than the commercial products. Commercial products made by extrusion have a crumbly texture. When teeth penetrate the product, the product cracks and splits; thus, the paraffin is not properly removed. In terms of infill density, 60% density for both glycerin 10% and glycerin 20% removed paraffin at more than 40% density because the net was formed more densely at 60% than at 40%. However, at 80% density, the removal efficacy decreased as the chew fell apart into chunks and split like the commercial product. The plaque removal efficiency was nearly equivalent between glycerin 10% and glycerin 20%, even though the dough formulation with glycerin 20% showed relatively low stress during chewing compared to that of 10% glycerin in the puncture test. Hence, it came to the conclusion that the 20% glycerin dough formulation was most suitable for making dental chews using 3D printing; moreover, 60% infill density was most effective for canine and premolar teeth in small dog breeds.

## 4 Conclusion

When making a dough formulation for starch-based dog dental chews, the addition of glycerin enhances printability. Corn starch with 10% and 20% glycerin was suitable for three-dimensional printing. All samples exhibited solid-like behavior in terms of their rheological properties, and G′ and G″ increased as the amount of glycerin increased. The moisture content of the dental chew with glycerin 20% was higher than that of the chew with glycerin 10% due to glycerin's water holding capacity; however, both samples had higher moisture content than that of the extrusion molding-based control. From the texture analysis results of the dental chew, dough with glycerin 20% placed less stress on teeth and gums because the hardness and breaking force for glycerin 10% were higher than those for glycerin 20%. Regarding the infill density, both the hardness and breaking force increased with higher infill levels. Compared to the control, all samples had a higher breaking force; otherwise, the samples had lower hardness. In the plaque removal efficacy test, 60% density was the most effective for both canine and premolar teeth because of the moderate size of the hole to scrape the tooth surface. Comparing the two teeth, 40% and 60% density dental chews were more effective in the canines, while the 80% density and control had more plaque reduction in the premolars. In conclusion, our study provides new insights into how 3D printing can be used to make dental chews with the different textures due to differences in infill levels and printing variables that can be consumed by dogs facing dental problems. Further research will be necessary to fit the dental chew structure through in vivo clinical studies, and the present study may provide valuable guidance for the use of 3D printing technology to modify dental chew products.

## Declarations

### Author contribution statement

Su Hyun Lee: Conceived and designed the experiments; Performed the experiments; Analyzed and interpreted the data; Contributed reagents, materials, analysis tools or data; Wrote the paper.

Hyun Woo Kim: Conceived and designed the experiments; Analyzed and interpreted the data; Contributed reagents, materials, analysis tools or data.

Hyun Jin Park: Analyzed and interpreted the data; Contributed reagents, materials, analysis tools or data.

### Funding statement

This work was supported by the Basic Science Research Program through the National Research Foundation of Korea (NRF) funded by the Ministry of Science, ICT & Future Planning (contract grant number NRF-2020R1I1A1A01073380).

### Data availability statement

Data included in article/supplementary material/referenced in article.

### Declaration of interests statement

The authors declare no conflict of interest.

### Additional information

No additional information is available for this paper.
